# Anti-borreliae efficacy of selected organic oils and fatty acids

**DOI:** 10.1186/s12906-019-2450-7

**Published:** 2019-02-04

**Authors:** Anna Goc, Aleksandra Niedzwiecki, Matthias Rath

**Affiliations:** Department of Infectious Diseases, Dr. Rath Research Institute, 1260 Memorex Dr., Santa Clara, CA 95050 USA

**Keywords:** *Borrelia* spp*.*, Oils, Fatty acids, Spirochetes, Persisters, Biofilm-like aggregates

## Abstract

**Background:**

*Borrelia* sp. is a causative pathogen of Lyme disease which has become a worldwide health concern. Non-toxic approaches especially directed toward latent persistent forms of this pathogen are desired. Lipids in the form of volatile and non-volatile oils, and fatty acids with proven anti-borreliae efficacy could become an additional support or an alternative for consideration in treatment approaches.

**Methods:**

In this study we investigated 47 lipids (30 volatile and non-volatile oils, and 17 fatty acids) of plant and animal origin against typical motile, knob/round-shaped persisters, and biofilm-like aggregates of *Borrelia burgdorferi s.s.* and *Borrelia garinii*, which are identified as pathogenic factors of Lyme disease in the USA and Europe, using direct microscopic counting and spectrofluorometric measurements.

**Results:**

Out of all examined lipids, 5 oils (Bay leaf oil, Birch oil, Cassia oil, Chamomile oil German, and Thyme oil) at or below 0.25%, and 3 fatty acids (13Z,16Z Docosadienoic acid, erucic acid, and petroselinic acid) at or below 0.75 mg/ml, showed bactericidal activity against typical motile spirochetes and knob/round-shaped persisters. Only Bay leaf oil and Cassia oil, including their major constituents, eugenol and cinnamaldehyde, showed to target biofilm-like aggregates of both tested *Borrelia* spp. at the same concentration*,* although with 20–30% eradication mark.

**Conclusion:**

Based on obtained results, volatile oils were more potent than non-volatile oils, and unsaturated fatty acids were more effective than saturated fatty acids. Among all tested oils, Bay leaf oil and Cassia oil, with their major components eugenol and cinnamaldehyde, seem to have the highest anti-borreliae efficacy.

## Background

Lyme disease (LD) is a multi-systemic zoonosis transmitted by ticks. According to the CDC, it has become the most common vector-borne disease in Europe and Northern America. In the USA alone the number of diagnosed LD cases reaches 300,000 annually [1]. The causative factor of this illness is a bacterium of genus *Borrelia* which is an invasive, host-dependent, semi-aerophilic, and slow-growing pathogen [2]. These features attribute to the long delays when diagnosing LD and, in many cases, a correct and precise diagnosis is a challenge. Currently, four predominant species are identified to cause LD: *Borrelia burgdorferi s.s.* and *Borrelia mayonii* (in North America) and *Borrelia afzelii* and *Borrelia garinii* (in Eurasia). All of the species can exist in three morphological forms. The vegetative (active) form is the typical corkscrew motile spirochetes. Under stress conditions, including antibiotic exposure in vitro, they have the ability to quickly transform into latent persistent forms such as knob/round-shaped bodies (forms) and/or biofilm-like aggregates [3–6]. Although not fully proven and accepted, it is believed that these latent forms might be one of the possible causes of the infection persisting in animal and human organisms [7–11].

The primary and conventional treatment for Lyme patients is based on antibiotics, and the “first choice” of β-lactams and tetracyclines are administered for up to 3–4 weeks. Randomized clinical trials showed that for the acute (early) stage of LD this approach can be nearly 90% effective, as opposed to the late and persistent stages of this disease, whose etiology, although is still not clearly explained, points to lingering persisting infection as one of the reasons [2, 12–14]. In addition, prolonged treatments with FDA-approved antibiotics are not recommended for pregnant women and patients with persistent LD (PTLDS, Post-Treatment Lyme Disease Syndrome), since their effectiveness has not been demonstrated, they are not cost-effective, and they are difficult to administrate, or may involve toxic side effects [1, 15–21]. Among patients suffering from chronic symptoms, approximately 20% of them remain without effective treatment [1]. The alternative non-antibiotic treatments are available but their efficacy has not yet been validated in clinical trials. One of the reasons might be the rather small number of compounds that have been studied thus far against *Borrelia* sp. [22–27]. However, naturally-derived substances, either in the form of the isolated active compounds, their metabolites, or plant-based extracts, have been a valuable source of non-synthetic and non-modified agents for human and animal health longer than the synthetic therapeutics [28–32]. Their potential as antimicrobials has been tested against an abundance of bacteria species. Lipid compounds have not yet been extensively studied as anti-borreliae agents. Their classification is rather unified and integrates a vast group of chemical compounds diverted in structure, properties, and function [33]. Lipids are commonly ascribed as hydrophobic viscous in ambient temperature compounds of plant, animal, or petrochemical origin. They encompass a class of molecules recognized as triglycerides, which are liquid in nature and called oils, as well fatty acids that are carboxylic acids with a long aliphatic chain, either saturated or unsaturated. Organic oils may be comprised in volatile or non-volatile groups with extensive use in industry, cosmetology, and food and medicine. Their remarkable applicability also evokes an interest as naturally occurring agents with broad antimicrobial characteristics [30–32].

Saturated or unsaturated fatty acids are the essential building blocks of other structurally complex lipids, and are important dietary sources of energy for animals. Thus, it is rational to study the biocidal potential of organic oils and fatty acids and validate their specific or general antimicrobial activity.

In this study, we tested 30 volatile and non-volatile organic oils, and 17 fatty acids against all known pleomorphic forms of *Borrelia burgdorferi s.s.* and *Borrelia garinii*. We evaluated their anti-borreliae effect using a “golden standard” method of direct counting and dark field microscopy, and the high throughput spectrofluorometric screening method to assess viability of the logarithmic phase (rich in typical motile spirochetes) and the stationary phase (rich in knob/round-shaped persisters) [20]. We also conducted quantitative testing of these lipids against biofilm-like aggregates of both studied *Borrelia* strains.

## Methods

### Test compounds

The test compounds and antibiotics with the purity between 90 and 98% according to the manufacturer, with the exception of the Greek sage oil (*Salvia triloba*) obtained from SunRose Aromatics, LLC (Morrill ME), the Bay leaf oil (*Pimenta racemosa*) obtained from Penn Herb Company, Ltd. (Philadelphia, PA), and 13Z,16Z docosadienoic acid purchased from Cayman Inc. (Ann Arbor, MI). Their selection was based on the literature review and potential applicability for commercial use as anti-borreliae agents. The stock solution (25–50 mg/ml) of each solid compound was prepared by suspending an individual test compound in DMSO and sterilizing with 0.22 μm syringe filtration. All liquid (stock) compounds were also sterilized with 0.22 μm syringe filtration. No difficulties were observed with dissolving the test lipids in DMSO.

### Test microorganisms

Two *Borrelia* species, *Borrelia burgdorferi* sensu stricto and *Borrelia garinii*, were tested in their three morphological forms: typical motile spirochetes, persisters/knob (round)-shaped forms, and biofilm-like aggregates. Low passage isolates of the B31 strain of *Borrelia burgdorferi* and the CIP103362 strain of *Borrelia garinii* were obtained from the American Type Culture Collection (Manassas, VA). The B31 strain is an isolate from *Ixodes dammini*, and the CIP103362 strain is an isolate from *Ixodes ricinus*. Both *Borrelia* sp. were prepared for testing as previously reported [6, 24]. Briefly, the cryo-stocks of both species were cultured in commonly used conditions, i.e., Barbour-Stoner-Kelly H (BSK-H) medium supplemented with 6% rabbit serum (Sigma, St. Louis, MO) without antibiotics at 33 °C with 5% CO_2_, in 15 ml polypropylene sterile test tubes, respectively. The homogeneous logarithmic culture (having only typical motile spirochetes/active form) of tested *Borrelia* sp. was obtained by maintaining inoculum in a shaking incubator at 33 °C and 250 rpm for 2–3 days. Stationary culture (enriched in knob/round-shaped cells/persistent forms) of tested *Borrelia* sp. was generated by maintaining inoculum in an incubator at 33 °C for 7–8 days. Biofilm-like aggregates of tested *Borrelia* sp. were prepared by incubation of inoculums in four-well chambers (BD Biosciences, Sparks, MD) coated with collagen Type I from rat tail for at least 1 week.

### Evaluation of the anti-borreliae effects of test lipids against typical motile spirochetes and knob/round-shaped persisters of *Borrelia* spp.

Testing was assessed using direct counting and spectrofluorimetry as previously described [26, 34]. Briefly, 1.8 ml sterile screw-cap test tubes or 96-well plates with 1 ml BSK-H medium, were inoculated with 2 × 10^6^–1 × 10^7^ cells/ml (logarithmic phase, i.e., 2–3 days culture or stationery phase, i.e., 7–8 days culture) and supplemented with the test compound in concentration ranging from 0.025 to 1.0% for liquid agents, and from 0.025 mg/ml to 1.0 mg/ml for solid agents. Controls were treated with DMSO (0.1–0.4%) or triple combination of antibiotics (daptomycin+cefoperazone+doxycycline) (0.03 mg/ml, 0.01 mg/ml each) since this combination of antibiotics showed to be effective against the logarithmic phase and the stationary phase of *Borrelia burgdorferi s.s.* [20]. Next, the tubes and plates were incubated at 33 °C with 5% CO_2_ up to 72 h. Assessment was done using high throughput spectrofluorometric screening and/or direct counting with a bacterial Petroff-Hausser counting chamber and dark field microscopy, supported by SYBR Green I/PI staining and a fluorescence microscope (Nikon, Eclipse E600), respectively. The excitation wavelength was set at 485 nm, and the fluorescence intensity at 535 nm (green emission) and 635 nm (red emission). Long-term sub-culture study was performed by transferring 50 μl of the stationary phase of *Borrelia* sp. culture, previously treated with test lipid and washed with fresh BSK-H medium, to 1 ml of fresh agent-free BSK-H medium for 14 days. The tubes were then incubated at 33 °C with 5% CO_2_ and bacterial proliferation was assessed using a bacterial Petroff-Hausser counting chamber with a dark field microscope and SYBR Green I/PI staining with a fluorescence microscope (Nikon, Eclipse E600) [34]. All experiments were conducted three times independently and each one in three replicates.

### Evaluation of anti-borreliae effects of the test lipids against biofilm-like aggregates of *Borrelia* spp.

Quantitative anti-borreliae effect of the test compounds against biofilm-like aggregates was assessed in four-well chambers coated with collagen Type I from rat tail (Sigma, St. Louis, MO) as described [5, 6, 26]. Briefly, 1 × 10^7^ cells/ml was inoculated into each sterile chamber filled with 1 ml BSK-H medium, and incubated for 1 week at 33 °C with 5% CO_2_, followed by 72 h of incubation with the test lipids. Control wells received DMSO (0.1–0.4%) or the triple combination of antibiotics (daptomycin+cefoperazone+doxycycline) (0.03 mg/ml, 0.01 mg/ml each). Eradication of biofilm-like structures was assessed by using the crystal violet (CV) staining method as previously reported [5]. Briefly, all wells were fixed with 500 μl of cold methanol-formalin (1:1) for 30 min. and stained with 1 ml of CV (0.1%) for 10 min. Next, the biofilms were carefully washed three times with 1 x PBS (phosphate-buffered saline), and 1 ml of methanol was added to each well to extract a dye which was measured at 595 nm using a spectrophotometer (Molecular Device, Spectra Max 340). In addition, all wells were fixed with 500 μl of cold formalin acetic acid mixture for 20 min., followed by staining with 200 μl of BacLight staining kit (Thermo Fisher, Waltham, MA) for 15 min. in the dark, according to the manufacturer’s recommendation. Pictures were immediately taken from untreated and treated mounted slides using a fluorescence microscope (Nikon, Eclipse E600). Earlier studies in our laboratory have documented a lack of antifungal carryover using this procedure [24]. All experiments were conducted three times independently and each one in three replicates.

### Statistical analysis

All data are presented as means ± SD (*n* = 3). The Student’s two-tailed t test was used to determine statistically significant differences set at 0.05 levels. Statistical analysis was performed using GraphPad software.

## Results

The bactericidal efficacy of selected lipid compounds against typical motile spirochetes (logarithmic phase) and knob/round-shaped persisters (stationary phase) of *Borrelia burgdorferi s.s.* (B31 strain) and *Borrelia garinii* (CIP103362 strain) are presented as the minimal bactericidal (MBC) concentrations in Tables 1 and 2. The results showed that among 30 tested oils, 22 expressed bactericidal activity against typical corkscrew motile spirochetes and knob/round-shaped persisters and the MBC values fluctuated between 0.15 and 1%. Out of all 17 tested fatty acids, 8 revealed the killing effect at the concentrations between 0.5 mg/ml and 1.0 mg/ml. The MBC values corresponded to each other for both tested *Borrelia* sp. The triple combination of antibiotics (daptomycin+cefoperazon+doxycycline) at the concentration of 0.03 mg/ml (0.01 mg/ml, each) was used as a positive control since this combination at this particular concentration was previously reported to be effective against both typical motile spirochetes and knob/round-shaped persisters of *Borrelia burgdorferi* [20, 26].

**Table 1:** Bactericidal efficacy of organic oils against logarithmic and stationary phases of *Borrelia burgdorferii s.s.* and *Borrelia garinii*

Tested oils	*Borrelia burgdorferi s.s.*		*Borrelia garinii*	
	Logarithmic phase MBC_90_ (%)	Stationary phase MBC_90_ (%)	Logarithmic phase MBC_90_ (%)	Stationary phase MBC_90_ (%)
Arnica oil	NS^a^	NS^a^	NS^a^	NS^a^
Avocado oil	NS^a^	NS^a^	NS^a^	NS^a^
Bay leave oil (*Pimenta racemosa*)	0.15	0.15	0.15	0.15
Birch (sweet) oil	0.25	0.25	0.25	0.25
Black pepper oil	1.0	1.0	1.0	1.0
Black seed oil	1.0	1.0	1.0	1.0
Borage oil	1.0	1.0	1.0	1.0
Cassia oil	0.15	0.15	0.15	0.15
Coconut oil	NS^a^	NS^a^	NS^a^	NS^a^
Coriander oil	1.0	1.0	1.0	1.0
Chamomile oil (German)	0.25	0.25	0.25	0.25
Chamomile oil (Roman)	1.0	1.0	1.0	1.0
Fennel oil	1.0	1.0	1.0	1.0
Grape seed oil	NS^a^	NS^a^	NS^a^	NS^a^
Greek Sage oil (*Salvia triloba*)	0.75	0.75	0.75	0.75
Helichrysum oil	1.0	1.0	1.0	1.0
Hyssop oil	0.75	0.75	0.75	0.75
Jasmin oil	0.75	0.75	0.75	0.75
Juniper oil	NS^a^	NS^a^	NS^a^	NS^a^
Laurel leaf (*Laurus nobilis*)	0.50	0.50	0.50	0.50
Myrrh oil	0.75	0.75	0.75	0.75
Nutmeg oil	0.75	0.75	0.75	0.75
Olive oil	NS^a^	NS^a^	NS^a^	NS^a^
Pumpkin seed oil	0.75	0.75	0.75	0.75
Pine needle oil	0.75	0.75	0.75	0.75
Safflower oil	NS^a^	NS^a^	NS^a^	NS^a^
Sunflower oil	NS^a^	NS^a^	NS^a^	NS^a^
Tarragon oil	1.0	1.0	1.0	1.0
Thyme	0.2	0.2	0.20	0.20
Turmeric oil	0.75	0.75	0.75	0.75
White camphor oil	0.75	0.75	0.75	0.75

*Abbreviations:* MBC_90_–minimal bactericidal concentration causing at least 90% of killing assessed by spectrofluorimetry after 72 h of treatment; ^a^–maximal tested concentration (i.e., 1%), NS–not susceptible/not satisfying MBC_90_ requirement at the maximal tested concentration

**Table 2:** Bactericidal efficacy of fatty acids against logarithmic and stationary phases of *Borrelia burgdorferii s.s.* and *Borrelia garinii*

Tested fatty acids	*Borrelia burgdorferi s.s.*		*Borrelia garinii*	
	Logarithmic phase MBC_90_ (mg/ml)	Stationary phase MBC_90_ (mg/ml)	Logarithmic phase MBC_90_ (mg/ml)	Stationary phase MBC_90_ (mg/ml)
Polyunsaturated				
Linolenic acid	NS^a^	NS^a^	NS^a^	NS^a^
Eicosapentaenoic acid	NS^a^	NS^a^	NS^a^	NS^a^
Docosahexaenoic acid	NS^a^	NS^a^	NS^a^	NS^a^
Linoleic acid	1.0	1.0	1.0	1.0
13Z,16Z Docosadienoic acid	0.5	0.5	0.5	0. 5
Monounsaturated				
Undecenoic acid	NS^a^	NS^a^	NS^a^	NS^a^
Palmitoleic acid	NS^a^	NS^a^	NS^a^	NS^a^
Oleic acid	1.0	1.0	1.0	1.0
Erucic acid	0.75	0.75	0.75	0.70
Petroselinic acid	0.75	0.75	0.75	0.75
Saturated				
Butyric acid	1.0	1.0	1.0	1.0
Undecanoic acid	NS^a^	NS^a^	NS^a^	NS^a^
Caprylic acid	NS^a^	NS^a^	NS^a^	NS^a^
Myristic acid	NS^a^	NS^a^	NS^a^	NS^a^
Palmitic acid	NS^a^	NS^a^	NS^a^	NS^a^
Stearic acid	NS^a^	NS^a^	NS^a^	NS^a^
Arachidic Acid	1.0	1.0	1.0	1.0
Dox + Dap+Cef	0.03	0.03	0.03	0.03

*Abbreviations:* MBC_90_–minimal bactericidal concentration causing at least 90% of killing assessed using spectrofluorimetry after 72 h of treatment, *Dox* doxycycline, *Dap* daptomycin, *Cef* cefoperazon, ^a^–maximal tested concentration (i.e., 1%), NS–not susceptible/not satisfying MBC_90_ requirement at the maximal tested concentration

**Fig. 1:**
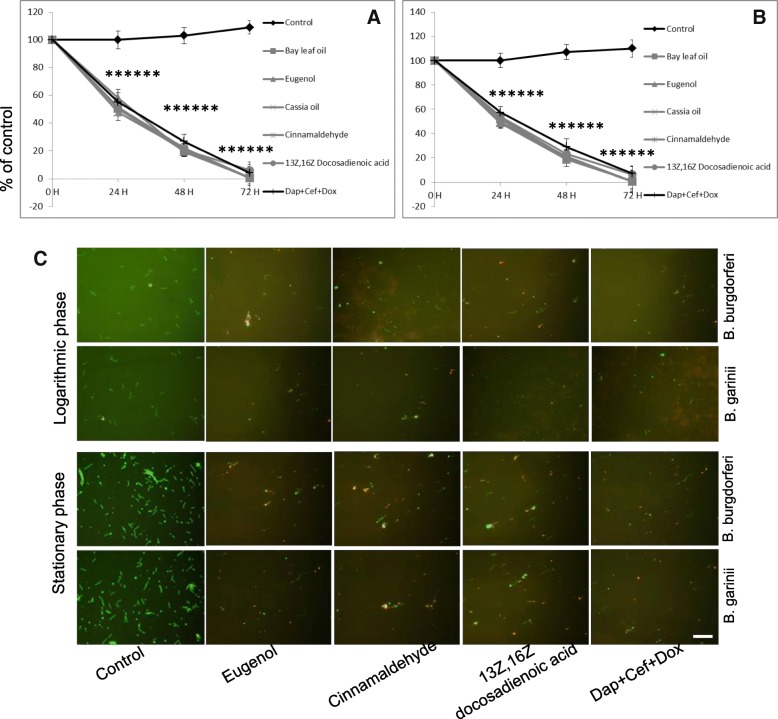
Kinetic evaluation of anti-borreliae effects of selected lipids. Time-dependent killing efficacy of test lipids (oils at 0.15% including their active ingredients at 0.125% concentration, 13Z,16Z-docosadienoic acid at concentration 0.5 mg/ml) against logarithmic and stationary phases of *Borrelia burgdorferi s.s.* (**a**) and *Borrelia garinii* (**b**) monitored up to 72 h using direct counting and fluorescence microscope; * *p* ≤ 0.001 compared to control. Representative live (green)/dead (red) merged images of logarithmic phase and stationary phase of *Borrelia burgdorferii s.s.* and *Borrelia garinii* (**c**) after 24 h incubation period with eugenol and cinnamaldehyde, as the active compounds of the most effective oils, 13Z,16Z-docosadienoic acid, and daptomycin+cefoperazone+doxycycline (Dap+Cef + Dox) as a positive control, taken at 200 x magnification, stained with BacLight dye; scale bar 50 μm

The results obtained from spectrofluorometric measurements have been further validated by direct counting using a dark field microscope and a fluorescence microscope for Bay leaf oil, Cassia oil and 13Z,16Z docosadienoic acid, all selected as agents with the highest anti-borreliae efficacy among all compounds tested in this study (Table 3). The results corroborated the data obtained from spectrofluorometric assay and, in addition, revealed that minimal inhibitory (MIC) values for Bay leaf oil and Cassia oil was 0.005%, and 0.025 mg/ml for 13Z,16Z docosadienoic acid. Moreover, bactericidal effect of these three lipids was time-dependent with the MBC_50_ value achieved after 24 h (Fig. 1). Also, in this set of experiments additional examination was performed to find out which of the active components in the most effective oils (Bay leaf oil, Birch oil, Cassia oil, Chamomile oil German, and Thyme oil) could be responsible for observed anti-borreliae effect. The results revealed that 0.125% of eugenol (present in Bay leaf oil), 0.25% of methyl salicylate (present in Birch [sweet] oil), 0.125% cinnamaldehyde (present in Cassia oil), 0.10 mg/ml of chamazulene (present in Chamomile oil German), and 0.20 mg/ml thymol (present in Thyme oil), could be the constituents with bactericidal activity in contrast to other active ingredients present in these oils that did not show such activity [35–39]. There were no significant differences in efficacy of these compounds between both tested *Borrelia* spp.

**Table 3:** Bacteriostatic and bactericidal efficacy of selected lipids and its active components against logarithmic and stationary phases of *Borrelia burgdorferii s.s.* and *Borrelia garinii*

Tested agents	*Borrelia burgdorferi s.s.*			*Borrelia garinii*		
	Logarithmic phase		Stationary phase	Logarithmic phase		Stationary phase
	MIC	MBC_90_	MBC_90_	MIC	MBC_90_	MBC_90_
Bay leaf oil	0.0005%	0.15%	0.15%	0.0005%	0.15%	0.15%
Eugenol	0.0003%	0.125%	0.125%	0.0003%	0.125%	0.125%
Birch oil	0.00075%	0.25%	0.25%	0.00075%	0.25%	0.25%
Methyl Salicylate	0.00075%	0.25%	0.25%	0.00075%	0.25%	0.25%
Cassia oil	0.0005%	0.15%	0.15%	0.0005%	0.15%	0.15%
Cinnamaldehyde	0.0003%	0.125%	0.125%	0.0003%	0.125%	0.125%
Chamomile oil	0.0075%	0.25%	0.25%	0.0075%	0.25%	0.25%
Chamazulene	0.01 mg/ml	0.10 mg/ml	0.10 mg/ml	0.01 mg/ml	0.10 mg/ml	0.10 mg/ml
α-bisabalol	NS^a^	NS^a^	NS^a^	NS^a^	NS^a^	NS^a^
Thyme oil	0.0005%	0.20%	0.20%	0.0005%	0.20%	0.20%
Thymol	0.02 mg/ml	0.20 mg/ml	0.20 mg/ml	0.025 mg/ml	0.20 mg/ml	0.20 mg/ml
γ-terpinene	NS^a^	NS^a^	NS^a^	NS^a^	NS^a^	NS^a^
13Z,16Z Docosadienoic acid	0.025 mg/ml	0.50 mg/ml	0.50 mg/ml	0.025 mg/ml	0. 05 mg/ml	0.50 mg/ml

*Abbreviations:* MIC–minimal bacteriostatic concentration causing at least 90% of growth inhibition assessed by direct courting using dark field microscopy after 72 h of treatment MBC_90_–minimal bactericidal concentration causing at least 90% of killing assessed using by direct courting using fluorescence microscopy after 72 h of treatment, ^a^–maximal tested concentration (i.e., 1%), NS–not susceptible/not satisfying MIC/MBC_90_ requirement at the maximal tested concentration

**Fig. 2:**
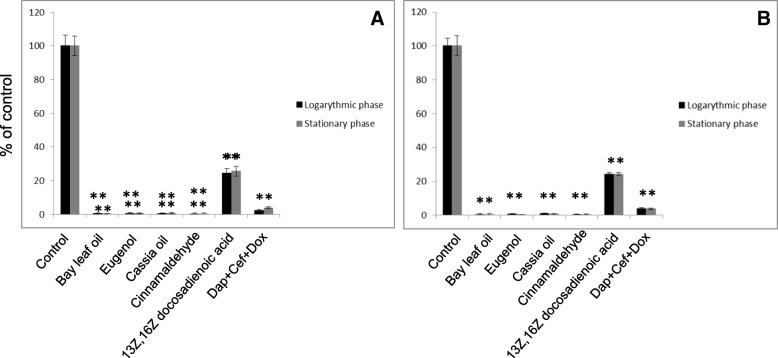
Estimation of repopulated spirochete forms of *Borrelia* spp. *Borrelia burgdorferi s.s.* (**a**) and *Borrelia garinii* (**b**) were treated with test lipid (oils at 0.15% including their active ingredients at 0.125% concentration, 13Z,16Z docosadienoic acid at concentration 0.5 mg/ml) for 72 h and transferred to fresh tubes containing BSK-H medium only. After 14 days of sub-culturing, the presence of typical motile spirochetes were determined by SYBR Green I/PI assay using direct counting and fluorescence microscope; Dap+Cef + Dox – daptomycin+cefoperazone+doxycycline (as a positive control); * *p* ≤ 0.001 compared to control

The results of the 14-day sub-culturing study were performed as described previously [26, 27] to verify the killing effect of the stationary phase of Bay leaf oil and Cassia oil, including their major active component cinnamaldehyde and eugenol, as well as 13Z,16Z docosadienoic acid, are presented in Fig. 2. They show that Bay leaf oil at 0.15% concentration, or 0.125% eugenol as well as Cassia oil, or 0.125% cinnamaldehyde, were able to repopulate the fresh BSK-H media with predominantly live cells in 0.1–0.3%. Treatment with the triple antibiotic combination (0.03 mg/ml) resulted in ~ 10–12% of viable cells as previously reported [26], and a 24% increase in the number of viable cells treated with 0.5 mg/ml 13Z,16Z docosadienoic acid. Direct microscopic counting confirmed the results obtained with the SYBR Green I/PI assay.

**Fig. 3:**
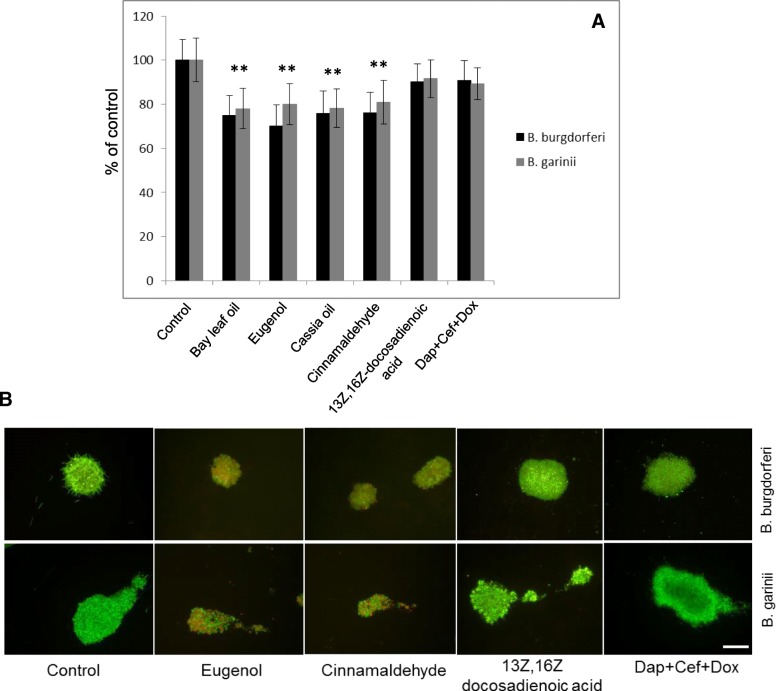
Susceptibility of *Borrelia burgdorferi s.s.* and *Borrelia garinii* biofilm-like aggregates to the test lipids. Biofilm-like aggregates grown on collagen-coated surface were treated with the test lipid (oils at 0.15% including their active ingredients at 0.125% concentration, 13Z,16Z docosadienoic acid at concentration 0.5 mg/ml), for 72 h (**a**). Next, the percentage of eradication of biofilm mass was determined by crystal violet staining method. * *p* ≤ 0.05 compared to control. Representative live (green)/dead (red) merged images of biofilms of *Borrelia burgdorferii s.s.* and *Borrelia garinii* (**b**) after 72 h incubation period with eugenol and cinnamaldehyde, as the active compounds of the most effective oils, 13Z,16Z-docosadienoic acid, and daptomycin+cefoperazone+doxycycline (Dap+Cef + Dox) as a positive control, taken at 200 x magnification, stained with BacLight dye; scale bar 50 μm

Evaluation of biofilm-like structures grown on collagen-coated surface did not show the eradication of the biofilm-like aggregates when treated with 13Z,16Z docosadienoic acid. This was in contrast to Bay leaf oil and eugenol, as well as Cassia oil and cinnamaldehyde, although biofilm decrease was at a range of ~ 20–30% (Fig. 3). Treatment with the triple antibiotic combination did not have any significant effect on *Borrelia* biofilm-like structures grown on collagen surface, as published earlier [26].

## Discussion

Naturally occurring compounds have been widely used in many aspects of human health for centuries [28–32, 40–43]. They have been extensively studied for their antimicrobial properties as well. Derived from a variety of biological sources such as plants, herbs, spices, and fruits, they have shown activity against a plethora of viruses, bacteria and fungi species, and are effective as food preservatives [44–47]. In addition, naturally-derived substances form the structural basis for developing new synthetic or semi-synthetic agents [29].

To date, most of the compounds investigated against *Borrelia* sp. belong to the polyphenols with only one study thus far that investigated volatile oils [25, 27]. The authors of this study concluded that the most active essential oils were oregano oil, cinnamon oil, and clove oil, displaying higher efficacy than daptomycin, an antibiotic shown to kill *Borrelia’s* persister. This study also indicated that oregano oil and its major constituent, carvacrol, are the most effective against the stationary phase of *Borrelia burgdorferi* with the ability of targeting biofilm-like clumps [27]. However, studies investigating other classes of lipid molecules such as fatty acids are lacking.

Our in vitro study expanded the pool of volatile oils to non-volatile oils and other classes of lipids such as fatty acids. The results indicate that out of 30 examined oils, Bay leaf oil and Cassia oil showed the highest anti-borreliae bacteriostatic and bactericidal effect, and moderate eradication effect of biofilm-like structures grown on collagen-coated surface. Additional evaluation of different concentrations of these two oils as well as their main active components, eugenol and cinnamaldehyde, displayed anti-borreliae effects in a time-dependent killing manner, which might point to a specific rather than non-specific mode of action. Interestingly, active ingredients in the most effective oils present in the highest amounts in these oils were responsible for observed anti-borreliae effect. One example is the difference between efficacy of Chamomile oil Roman and Chamomile oil German with the latter being more potent but also containing a higher amount of chamazulene [37]. In regards to fatty acids, out of the 17 tested, 13Z,16Z docosadienoic acid showed most bacteriostatic and bactericidal effect. In contrast to the oils, none of these compounds significantly affected biofilm-like structures of *Borrelia* spp. when used at a concentration sufficient to kill the logarithmic phase and the stationary phase of bacteria.

In order to find out whether viable cells can regrow after a 14-day period in the absence of test lipids, we conducted sub-culturing experiments using the lipid-treated stationary phase by transferring its 1/20 portion into fresh culture medium. This study revealed that both Bay leaf oil and Cassia oil and their main ingredients (eugenol and cinnamaldehyde) are effective biocides, since no regrowth of viable cells was detected after a 14-day subculture. This was in contrast to 13Z,16Z docosadienoic where ~ 24% regrowth was observed. The 0.03 mg/ml (0.003%) triple antibiotic combination (daptomycin, doxycycline, and cefoperazone) was effective against typical motile spirochetes and knob/round-shaped persisters, but not biofilm-like aggregates grown on collagen surface, and treatment with the triple antibiotic combination resulted in ~ 11% regrowth of viable cells, as reported previously [26].

Antibacterial efficacy of Bay leaf oil (*Pimenta racemosa*) often misled with Laurel leaf (*Laurus nobilis*), Birch oil (*Betula lenta*), and Cassia oil (*Cinnamomum cassia*) misled with Cinnamon oil (*Cinnamomum zeylanicum*), in contrast to other volatile oils included in this study, e.g. Chamomile oil German (*Matricaria chamomilla),* Chamomile oil Roman *(Chamaemelum nobile*), Laurel leaf (*Laurus nobilis*), Thyme oil (*Thymus vulgaris*), etc., have not been extensively studied by other research groups against gram-positive and gram-negative bacteria, and there is no record about their anti-borreliae efficacy [48–52]. However, it is worth mentioning that Feng, et al.*,* recently reported about the profound efficacy of Clove oil (*Syzygium aromaticum*), Cinnamon oil (*Cinnamomum zeylanicum*), and Oregano oil (*Origanum vulgare*) with its main component carvacrol, against the stationary phase of *Borrelia burgdorferi* at the MBC_90_ value established to be at or above 0.1%.

Efficacy of active ingredients of the oils tested in this study and shown to be the most effective against *Borrelia burgdorferi s.s.* and *Borrelia garinii* such as eugenol, methyl salicylate, cinnamaldehyde, chamazulene, and thymol, against resistant and non-resistant gram-positive and gram-negative bacteria has been demonstrated more broadly [30, 31, 39, 48–54]. Interestingly, it was chamazulene and not α-bisabalol, which are both present in Chamomile oil German, to be responsible for anti-borreliae effect. Reported MBC values depend on the species and their resistance, and range from 0.01 to 1.9%, with the noticeable pattern of higher bactericidal concentrations being effective against their latent forms, clinical isolates and resistant strains [52]. MBC values obtained in our study have corroborated the ranges of those already reported. As well, the same efficacy was found to be against the logarithmic phase (enriched in typical motile spirochetes) and the stationary phase (enriched in persisters). Eugenol and cinnamaldehyde, as representatives of phenylpropanoids, were found to be especially effective against typical motile, knob/round-shaped persisters, and the micro-colonies of studied *Borrelia* spp. as presented in Figs. 1, 2 and 3. Interestingly, these compounds are major parts of many essential oils and powdered extracts that showed to be active against a plethora of other microbials such as viruses, yeasts, molds, and gram-positive and gram-negative bacteria, including resistant strains and biofilms. It is also worth mentioning their anti-oxidant properties and their commercial applications in food flavorings, cosmetics, dentistry, and dermal drug delivery [55, 56]. Moreover, eugenol and cinnamaldehyde have recently become a focus of interest due to their anti-cancer and anti-inflammatory potential [57, 58]. They have attracted a lot of interest for their bioactivities and applicability in agriculture as well [50, 55, 56]. Furthermore, these molecules are known to react with some enzymes and/or receptors consequently generating diverse therapeutically relevant pharmacological functions. Thus, these compounds are very promising candidates for versatile applications. Although the metabolism and pharmacokinetics of these compounds in humans have been studied, their therapeutic usage still remains to be further explored, and likewise research on chemical syntheses and modifications have been carried out to obtain compounds with more effective bioactivity. Finally, it is important to note that the effects of these two compounds on bacterial morphology has been observed, rendering possible action mostly towards their cell and mitochondrial membrane and the function of key survival-related metabolic enzymes [53, 54]. Thus, being hydrophobic in nature they are effective biocidal agents. However, cellular adaptation to these oils has not yet been reported, although it cannot be excluded.

There are numerous studies evaluating antibacterial properties of saturated and unsaturated fatty acids, but none relate to their anti-borreliae effects. Moreover, fatty acids operating as key components of antimicrobial food additives inhibiting the growth of undesirable microorganisms are well documented [40, 41, 59–61]. Besides the common fatty acids, it has been reported about their derivatives showing potent antimicrobial activities [41]. Interestingly, the antibacterial efficacy of fatty acids is usually ascribed to long-chain unsaturated fatty acids rather than generally less effective long-chain saturated fatty acids [41, 62]. It is also worth mentioning that long-chain unsaturated fatty acids are biocidal against well-known pathogens such as Methicillin-resistant *Staphylococcus aureus, Helicobacter pylori, Mycobacteria* sp., *Bacillus* sp. and *Streptococcus* sp. [62–66].

Based on our study we concluded that unsaturated fatty acids were more potent than saturated fatty acids against *Borrelia* spp. Moreover, the most effective were the fatty acids that have 18–22 carbons such as polyunsaturated 13Z,16Z docosadienoic acid (omega 6, C_22_), as well as monounsaturated erucic acid (omega 9, C_22_) and petroselinic acid (omega 12, C_18_). Interestingly, the unsaturated fatty acids that are classified as omega 6, 9, and 12 were more potent than omega 3. Their molecular target and mode of action still remains unknown, even though there have been several reports that point to the cell membranes as a primary target [67]. Greenway, et al.*,* [68] suggested that fatty acids with 18 carbons may have a surfactant-type action by increasing membrane permeability. Others have suggested auto-oxidation products of unsaturated fatty acids that could be toxic to bacteria [69]. In addition, extensive pharmacokinetic, pharmacodynamics, and toxicological studies are warranted before extrapolating their applicability from bench to bedside.

## Conclusions

Collectively, this study documents in vitro efficacy of a range of lipid compounds against active and latent forms of *Borrelia burgdorferi* sensu *lato* and indicate their being a promising non-synthetic alternative for combating these bacteria. These and earlier reported data by other groups support the hypothesis that organic oils in particular may be valuable and cost-efficient antimicrobials, and could be especially beneficial as topical or oral intervention in the case of other limited resources. Although further in vivo and human studies with attention paid to their safety are warranted, the organic oils could also become important antimicrobial agents improving the outcome of traditional treatments, and/or as an alternative option to conventional approaches.

## Abbreviations

CDC: Centers for Disease Control and Prevention
Cep: Cefoperazone
CV: Crystal violet
DAP: Daptomycin
DMSO: Dimethyl sulfoxide
Dox: Doxycycline
LD: Lyme disease
MBC: Minimal biocidal concentration
MIC: Minimal inhibitory concentration
PBS: Phosphate buffered saline
PI: Propidium iodide
PTLDS: Post-Treatment Lyme Disease Syndrome
